# A162 RARE PRESENTATION OF EXTRANODAL NK/T-CELL LYMPHOMA INVOLVING STOMACH AND EYE: CASE REPORT AND LITERATURE REVIEW

**DOI:** 10.1093/jcag/gwac036.162

**Published:** 2023-03-07

**Authors:** H J Kim, L Tam, W Xiong, G Rosenfeld

**Affiliations:** 1 Gastroenterology; 2 University of British Columbia, Vancouver, Canada; 3 Pathology, University of British Columbia, Vancouver, Canada

## Abstract

**Background:**

Extranodal NK/T-cell lymphoma (ENKTL) is a rare and aggressive form of non-Hodgkin lymphoma. ENKTL are predominantly localized to nasal and upper aerodigestive sites, but extranasal involvement including gastrointestinal tract are rarely seen. Small and large intestines are primary sites of gastrointestinal ENKTL. Gastric involvements are exceedingly rare accounting for less than 5% of all gastrointestinal ENKTL.

**Purpose:**

We present a literature review on gastrointestinal ENKTL and a case report of gastrointestinal bleed secondary to ENKTL involving stomach and left orbit.

**Method:**

Case report and literature review.

**Result(s):**

33-year-old female was admitted to a tertiary hospital with 3-week history of epigastric pain and left periorbital swelling. Abdominal CT showed edema and thickening of gastric folds. Head CT showed grossly enlarged left lateral rectus muscle and periorbital soft tissue swelling suggestive of left orbital pseudotumor. Esophagogastroduodenoscopy revealed multifocal Forrest classification II-C ulcerations throughout her stomach and duodenum. Biopsies from stomach showed gastric mucosa with extensive infiltration by an atypical lymphoid cell population. Immunohistochemistry demonstrated high grade lymphoid cells with uniform expression of CD2, CD3, CD30, CD56, TIA1, perforin and granzyme B. EBV-encoded small RNA in-situ hybridization (EBER ISH) was strongly positive. Findings were consistent with gastric ENKTL. Left orbital biopsy revealed similar morphology and phenotypic features consistent with concurrent ENKTL involvement of her orbit. Patient was initiated on intravenous corticosteroids, but unfortunately developed hemorrhagic shock secondary to gastrointestinal bleeding from gastric ulcer and passed away.

Gastrointestinal ENKTL is a rare presentation of a rare disease. Gastric involvement is especially rare and described only in few case reports. Gastrointestinal ENKTL are often initially asymptomatic but can progress to abdominal pain, bleeding and even bowel perforation. Due its nonspecific clinical features and rarity, diagnosis can be difficult and requires careful examination by an experienced pathologist. This aggressive lymphoma is characterized by positive CD2, CD3, CD30, CD56, TIA, granzyme B, perforin and EBER ISH. Optimal treatment approach remains unclear due to lack of prospective clinical studies. Currently, treatment modalities used for other lymphomas including radiotherapy and non-anthracycline-based chemotherapy are used. Despite treatment, prognosis is grim with median overall survival period of 7-8 months.

**Image:**

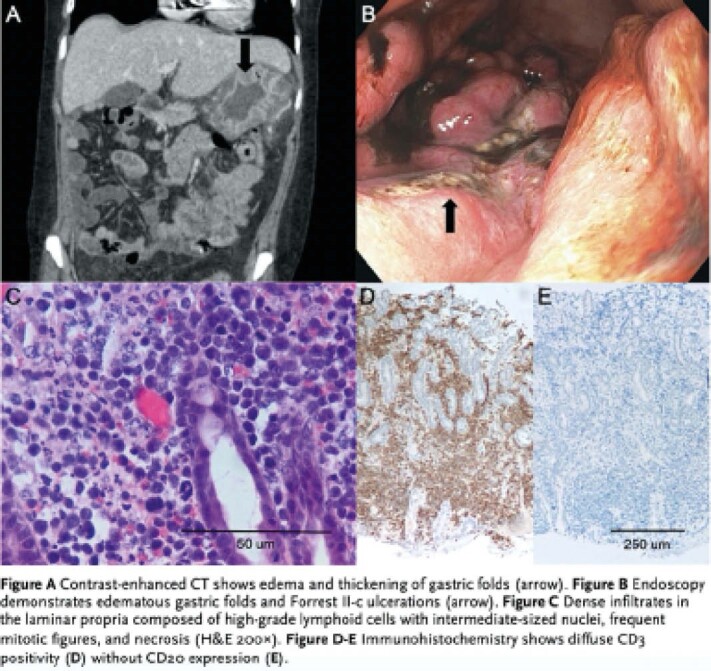

**Conclusion(s):**

We present a case of gastric and orbital ENKTL with gastric ulcer bleeding. Gastric ENKTL disease is a rare presentation of a rare disease. Due to non-specific clinical features, diagnosis is often difficult and relies on careful pathology examination by experienced pathologist. Prognosis is poor without optimal treatment approach due to rarity of disease and lack of validated data.

**Please acknowledge all funding agencies by checking the applicable boxes below:**

None

**Disclosure of Interest:**

None Declared

**INFLAMMATORY BOWEL DISEASES:**

MECHANISMS AND TREATMENTS

